# Neuromechanical Model of Rat Hindlimb Walking with Two-Layer CPGs †

**DOI:** 10.3390/biomimetics4010021

**Published:** 2019-03-01

**Authors:** Kaiyu Deng, Nicholas S. Szczecinski, Dirk Arnold, Emanuel Andrada, Martin S. Fischer, Roger D. Quinn, Alexander J. Hunt

**Affiliations:** 1 Department of Mechanical and Aerospace Engineering, Case Western Reserve University, Cleveland, OH 44106, USA; nss36@case.edu (N.S.S.); rdq@case.edu (R.D.Q.); 2 Institute of Zoology and Evolutionary Research, Friedrich-Schiller University Jena, Erbertstr. 1, 07743 Jena, Germany; d.arnold@uni-jena.de (D.A.); emanuel.andrada@uni-jena.de (E.A.); martin.fischer@uni-jena.de (M.S.F.); 3 Department of Mechanical and Materials Engineering, Portland State University, Portland, OR 97207, USA; ajh26@pdx.edu

**Keywords:** synthetic nervous system, rat, rhythm generator, pattern formation, muscle synergies

## Abstract

This work demonstrates a neuromechanical model of rat hindlimb locomotion undergoing nominal walking with perturbations. In the animal, two types of responses to perturbations are observed: resetting and non-resetting deletions. This suggests that the animal locomotor system contains a memory-like organization. To model this phenomenon, we built a synthetic nervous system that uses separate rhythm generator and pattern formation layers to activate antagonistic muscle pairs about each joint in the sagittal plane. Our model replicates the resetting and non-resetting deletions observed in the animal. In addition, in the intact (i.e., fully afferented) rat walking simulation, we observe slower recovery after perturbation, which is different from the deafferented animal experiment. These results demonstrate that our model is a biologically feasible description of some of the neural circuits in the mammalian spinal cord that control locomotion, and the difference between our simulation and fictive motion shows the importance of sensory feedback on motor output. This model also demonstrates how the pattern formation network can activate muscle synergies in a coordinated way to produce stable walking, which motivates the use of more complex synergies activating more muscles in the legs for three-dimensional limb motion.

## 1. Introduction

Neural circuits in the spinal cord, called central pattern generators (CPGs), generate neural oscillations and control rhythmic movements. These CPGs are responsible for repetitive motions such as walking [1,2], swimming [3], and breathing [4]. However, it is still unclear how CPGs contribute to motoneuron activations and muscle forces. Understanding this relationship will contribute to many fields, including understanding the effects of disease on locomotor circuits, repair and rehabilitation of injuries [5], and even controlling robots. There are many parallels between robotic and neurobiological walking controllers [6]. However, there are still many questions about how neurobiological systems produce dynamic and robust walking. For example, little is known about the details of motoneuron activity when animals perform steady walking, as it is difficult to record from individual neurons of live, intact animals as they move freely. Computational modeling is a useful tool to test how neural circuits give rise to walking [7]. For this research we built an intact rat walking model to test hypothetical networks that connect CPGs to motoneurons.

Previously, we developed a synthetic nervous system (SNS) that applies the neurobiological hypothesis of CPGs as a primary component of the controller for walking. We successfully showed that the reduced pattern generating circuit is sufficient for performing forward walking in a rat model and a dog robot [8,9]. However, in the CPG model we implemented in that previous network, the rhythm generating oscillators were directly connected to motoneurons. Thus, any excitation of the CPGs altered step cycle timing and motoneuron activation amplitude simultaneously [10]. This makes it difficult to modify rhythm timing and muscle force independently, as animals frequently do.

Recent animal and neural modeling experiments have demonstrated there may be two separate structures in locomotor spinal circuits. Studies of the fictive locomotor activity of decerebrate cats [11,12,13,14] revealed that: (1) afferent stimulation can advance or prolong phase switching within the ongoing step cycle without changing the following step cycle’s timing, and (2) in instances where motoneuron bursts were absent, subsequent motoneuron bursts reappear at the time they would be expected, had the deletion not occurred. These so-called “non-resetting deletions” were also found in motoneuron intracellular recording studies during scratch reflex activity in turtles [15] and mice [16,17]. In fact, Zhong et al. [17] revealed that nearly all (over 90%) of the deletions were non-resetting in mice. These two experimental results suggest that the CPG should have an internal structure that can ‘‘store’’ the locomotor cycle rhythm and phase, even when the output of that structure is not directed to the motoneurons.

Based on these studies, Rybak and colleagues [13,14,18,19,20] hypothesized a computational model of a two-layer CPG containing a half-center rhythm generator performing a “clock” function, and an intermediate pattern formation network that distributes and coordinates motoneuron pool activity. This hypothesized two-layer CPG allows separate control of walking rhythm timing and the activity pattern of motoneurons during locomotion. In this paper, we apply a two-layer CPG neural model for control of a physics-based biomechanical model and test the robustness of this neuromechanical system to neural and mechanical perturbations.

Researchers working in parallel have done some similar work. Markin’s group developed a neuromechanical model of cats that includes a two-layer CPG and a musculoskeletal model of cat hind limbs that generates motion-dependent afferent input to the neural model [20]. However, there are differences in the modeling approach taken that distinguish our model and, in particular, our results from those of Markin’s work.

The method in which the pattern formation layer activates the multitude of muscles in the limb during walking is still unknown; however, a leading theory involves the use of muscle synergies [12,20,21,22,23,24]. It is theorized that the central nervous system (CNS) produces a wide range of motor behaviors by co-activating groups of muscles using similar activation patterns in space or time [21,23,25]. There are two types of muscle synergies [24,25]: (1) “synchronous synergies”, which activate all muscles at the same time with no temporal delay; and (2) “time-varying synergies”, which produce patterns with a temporal profile of weighting coefficient for each muscle. The muscle synergy theory works well with the two-layer CPG model as it allows for the modification and modulation of different muscle synergies through the pattern formation layer without affecting the overall timing of the step cycle kept by the rhythm generating structure. For example, in 2012, Markin et. al. [22] identified the possible motoneuronal and muscle synergies operating during both fictive and real locomotion. They determined muscle activation patterns in fictive locomotion were consistent with activation patterns seen in real locomotion for single-joint muscles. However, this consistency did not hold for biarticular muscles. Later in 2016, in collaboration with Shevtsova, they constructed a 10 degrees of freedom biomechanical model of a cat hindlimb [20]. By implementing the identified muscle synergies in the pattern formation network of the CPG, and using the pattern formation network as synergy distributor, they successfully reproduced the patterns of biarticular muscle activities during fictive locomotion and validated muscle behaviors during deletions. This research suggests that muscle synergy is a viable solution to simplify the control problem in complex situations.

The research described in this paper applies the two-layer CPG hypothesis to a neuromechanical model of the rat hindlimb during locomotion. Here, we aim to test whether the rat model simulation could match existing experimental data by separating the control of motoneuron timing and magnitude. To reach this goal, we expanded our previous model to include a new, leg-level rhythm generator that helps coordinate the timing of the pattern generating circuits. We demonstrate the success of this expansion in several ways. First, we compare the neural behavior of the CPG of the deafferented neuron model with that of the decerebrate cat fictive locomotion experiments. After reproducing the animal behavior with our simplified neural circuits, we apply this structure to the intact, neuromechanical rat model, and measure the impact of sensory feedback in the simulation. In order to examine whether muscle synergies could reduce the complexity of the circuit design, two pattern formation groups are used to activate the six muscles in the hindlimb, one activating hip muscles while the other co-activates knee and ankle muscles. Our results show that the two-layer CPG successfully replicates non-resetting deletions and produces stable walking with a simple muscle synergy. This two-layer CPG model of locomotion demonstrates complex interdependencies of mechanics, sensory feedback, and rhythm generation during phase resetting and non-phase resetting deletions of locomotor rhythms.

## 2. Methods

**Figure 1:**
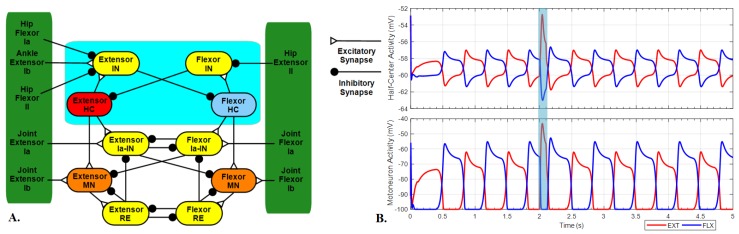
(**A**) Single limb joint control network. (**B**) Neuron activities of half-center neurons and motoneurons without feedback. Excitatory stimulus with a current of 2 nA applied to an extensor half-center neuron from 2 to 2.1 s (blue area). EXT: Extensor; FLX: Flexor; HC: Half-center neuron; IN: Interneuron; MN: Motoneuron; RE: Renshaw cell.

Our previous SNS for rat hindlimb locomotion used isolated subnetworks to control individual limb joints [8]. It successfully reproduced sagittal plane forward walking, using proprioceptive feedback (type Ia, Ib, and II) to coordinate step timing. Those isolated subnetworks (Figure 1A) are composed of a half-center oscillator, motoneurons and interneurons. The cyan box is the half-center oscillator. The half-center neurons possess persistent sodium channels and mutually inhibit each other via interneurons. The half-center oscillator can generate repetitive oscillations without external inputs to maintain the switches between extension and flexion. The green area includes all the proprioceptive feedback, which play an important role during rat walking. Feedback from the entire limb is applied directly to the CPG of each joint in the form of hip flexor Ia and II, hip extensor II, and ankle extensor Ib feedback to coordinate stance–swing timing [9]. Extensor and flexor Ia and Ib feedback from each joint feed directly back onto the joint control through Ia interneurons or directly onto the motoneuron to mediate muscle velocity and force production. Details about feedback and swing–stance relationship are derived from a variety of experiments and are outlined in Hunt et al. [26]. For this study, we are extending the previous work by layering on additional neural circuitry while maintaining the previous feedback pathways that mediate the coordination between joints.

In the previous, single layer model, the CPG synapsed directly onto the motoneurons. As sensory feedback shapes CPG behavior and period, this structure is sufficient to produce steady coordinated walking. However, any perturbation to the CPG system results in simultaneous changes both in step timing and muscle force magnitude, which does not allow for controlling force generation and rhythm separately. To demonstrate this, we applied an excitatory stimulus to an extensor neuron of the half-center to simulate a sudden perturbation (Figure 1B). To eliminate other influences, we removed all sensory feedback from the network, the only variable is the excitatory stimulus. The motoneuron successfully generates more muscle force to balance this influence, but the step timing generated by the CPG is also shifted, which could not replicate the non-resetting deletion phenomena. Also, as our model implemented separate limb joint controllers, the communication between left and right hindlimbs becomes complex, and these perturbations lead to an unintended change in gait.

### 2.1. Model Description

**Figure 2:**
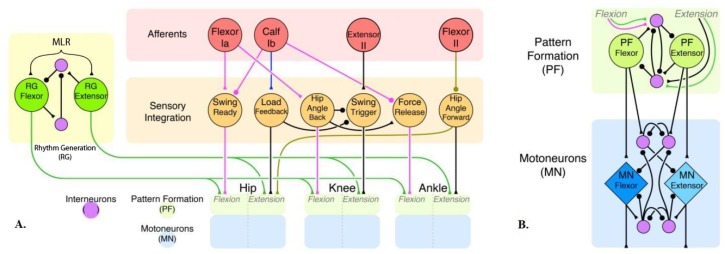
(**A**) Hypothetical synthetic nervous system for a single limb. (**B**) Limb joint control network that forms active pattern of motoneurons. Coordinating pathways are inhibitory (filled circles) or excitatory (filled triangles). Pathways inspired by biological research are indicated by colored synapses of magenta, blue, and brown [26]. Hypothesized pathways are in black. MLR: Midbrain locomotor region.

The conceptual neural circuit for a single limb (Figure 2A) builds on our previous models [8,9]. Each joint has an independent control network (Figure 1A and Figure 2B) that manages the pattern activity of motoneurons. That is the extent of our previous work. In this new model, the half-center oscillators act as pattern formation layers, which are only responsible for reshaping the timing signals and managing motoneuron activation. We have added a rhythm generator network as a four-neuron half-center oscillator to generate swing–stance rhythm. This network synapses onto every pattern formation layer in the limb, helping to regulate timing. In our model, we use relatively few non-spiking neurons to represent populations of spiking neurons. The network consists of leaky-integrator neuron models. The dynamics of which are described by the differential equations [27]:

$$C\frac{dV}{dt}=G\left(E_{rest}-V\right)+\sum \;G_{syn}\left(E_{syn}-V\right)+G_{Na}\left(E_{Na}-V\right)\cdot m\cdot h$$

$$\frac{dm}{dt}=\frac{m_{\infty }-m}{\tau_{m}},\;\frac{dh}{dt}=\frac{h_{\infty }-h}{\tau_{h}}$$

$$z_{\infty }=\frac{1}{1+A_{z}\cdot \exp\left(S_{z}\;\left(V-E_{z}\right)\right)}$$

$$G_{syn}=\{\begin{array}{c} 0,\;if\;V_{pre}<E_{lo}\\ g_{syn}\frac{V_{pre}-E_{lo}}{E_{hi}-E_{lo}},\;if\;E_{lo}<V_{pre}<E_{hi}\\ g_{syn},\;if\;E_{hi}<V_{pre} \end{array},$$

where V is the membrane voltage of the neuron, C is the membrane capacitance, $E_{rest}$ is the resting potential of the neuron, t is the time variable, and *E* stands for a constant reference voltage (i.e., reversal potential). $G\left(E_{rest}-V\right)$ is the leak current, $G_{syn}\left(E_{syn}-V\right)$ are currents due to synaptic connections, and $G_{Na}\left(E_{Na}-V\right)$ is a persistent sodium current present in rhythm generating neurons with voltage-dependent channel activation and inactivation described by m and h, respectively. z Represents either m or h, and time constant (τ), factor (*A*), slope (*S*) and reversal potential (*E*) are constant parameters, specific to m or h. For interneurons, these parameters are set to 0, and there are no dynamic sodium currents. The parameters $g_{syn}$, $E_{lo}$, and $E_{hi}$ are constants representing the synapse’s maximum synaptic conductance, its lower threshold, and its upper threshold, respectively. $V_{pre}$ here is the presynaptic neuron voltage. Details of neuronal and synaptic parameter can be found in Appendix A (Table A1 and Table A2).

**Figure 3:**
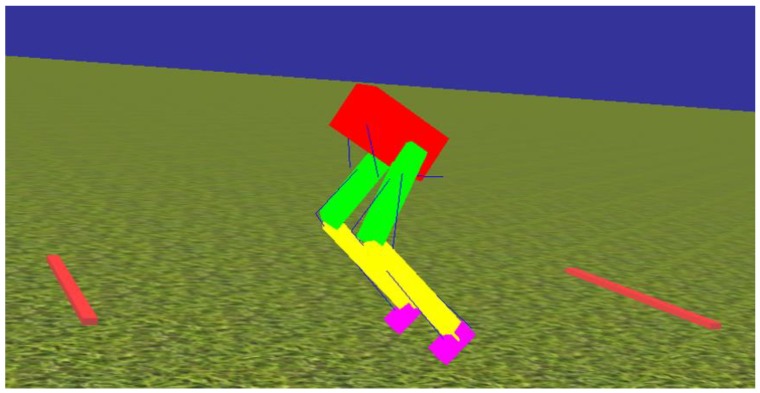
Biomechanical model of a rat hindlimb. Motion is constrained in the sagittal plane with hinge joints. The two bars on the ground support the body.

All the modeling and simulation was performed in Animatlab [28]. The biomechanical model reconstruction is limited to motion in the sagittal plane for the rat hindlimbs, resulting in six actuated degrees of freedom, three for each hindlimb (hip, knee, and ankle), and three unactuated degrees of freedom (the forward and vertical translation, and pitch angle of the pelvis). To improve simulation and calculation speed, the biomechanical model was simplified by reducing the complexity of segment shapes (Figure 3). Instead of loading rat bone files scanned from X-ray images as previously described [8,26], the pelvis (red), femur (green), tibia (yellow), and foot (purple) were constructed with simple box models. This resulted in the simulation running four times faster. The length, weight, and density of the bones, and the insertion points and properties of muscle are the same as in our previous biomechanical model. Similar to our previous work [8], two bars, one in front and one in back, support the body during simulated walking experiments.

**Figure 4:**
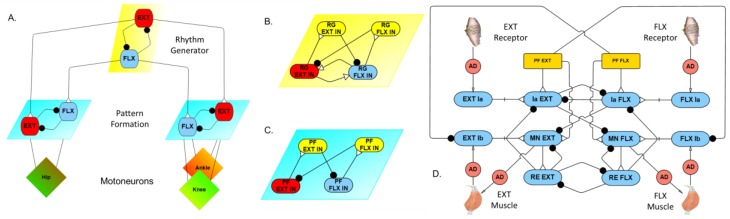
Synthetic nervous system. (**A**) Single limb control hierarchy. (**B**) Rhythm generator configuration. (**C**) Pattern formation network. (**D**) Sensory-motor network. AD: Adaptor; EXT: Extensor; FLX: Flexor; IN: Interneuron; PF: Pattern formation; RE: Renshaw cell; RG: Rhythm generation.

The new neural network is composed of a three-level hierarchy (Figure 4A). The top layer is the rhythm generator layer (Figure 4B), which is located in the spinal cord in vertebrates. We modeled our rhythm generator similar to the one reported by Zhang et al. [29], with two interacting neurons coupled by mutual excitation (weak connection) and inhibition (via inhibitory interneurons). The endogenous rhythmogenic properties of rhythm generator neurons are generated using persistent sodium channels [13]. The rhythm generator defines the rhythm of locomotion and the general timing of the extensor and flexor phases. The second layer is the pattern formation network (Figure 4C) located in the lower thoracic and lumbar segments of the spinal cord. The knee and ankle share one pattern formation network. The last layer is the motoneuron layer (Figure 4D); this layer mediates the magnitude of motoneuron activation with feedback, it is connected to muscles and responsible for generating muscle force.

The pattern formation layer reshapes the swing–stance timing signals received from the rhythm generator. According to Rybak’s model [13,14,18,19], the pattern formation layer distributes the information to synergistic motoneuron pools. The implementation of muscle synergies in decerebrate cat models [20,22] motivated us to apply such muscle synergy into our simulation model. In our previous work, activation of the knee and ankle muscles was found to be in tandem [8]. Therefore, we implemented a knee–ankle synergy which coordinates extensor and flexor muscles in these two joints, and so they share a pattern formation network.

### 2.2. Model Validation

Before implementing the two-layer CPG structure into the full neuromechanical rat model, it was first tested in isolation to see if the deafferented model could reproduce results from the decerebrate cat [12] and mouse [17]. In these experiments, the rhythm generator is connected to the pattern formation network, which is, in turn, connected to a single pair of flexor and extensor motoneurons. The whole network is isolated with no sensory feedback.

In the first test, the rhythm generator undergoes external stimulation. The stimulus is applied to both extensor and flexor neurons of the rhythm generator to simulate a descending command from the midbrain locomotor region (MLR). The same current is applied to both the extensor neuron and flexor neuron, so the extensor and flexor period are identical. The second set of tests examines the influence of external currents on the pattern formation layer. For this test we applied two stimuli to the model, one stimulus is shorter than one step cycle and the other is longer than one step cycle. This test investigates how the two-layer CPG reacts to external currents and if it reproduces the findings on the decerebrate cat that we mentioned in the Introduction section.

**Figure 5:**
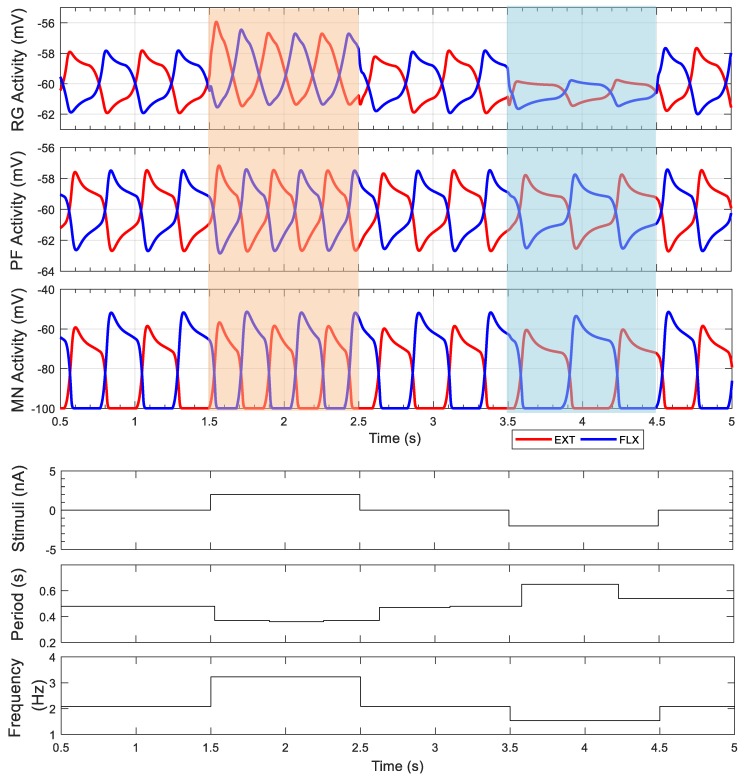
Rhythm generator performance under different conditions. Excitatory stimulus applied from 1.5 to 2.5 s (orange area) with a current of 2 nA. Inhibitory stimulus applied from 3.5 to 4.5 s (blue area) with a current of −2 nA. Both stimuli applied to extensor and flexor rhythm generator neurons simulate descending signals from the midbrain locomotor region. The simulation startup transient, corresponding to the first 0.5 s is not shown. EXT: Extensor; FLX: Flexor; MN: Motoneuron; PF: Pattern formation; RG: Rhythm generation.

**Figure 6:**
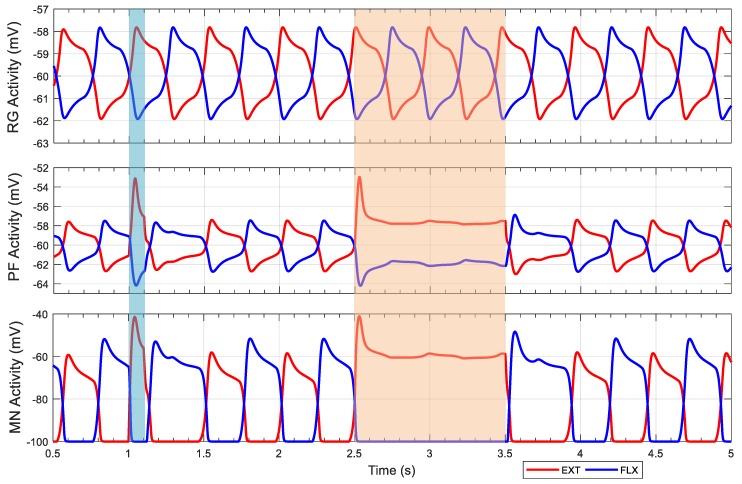
“Memory” function of a two-layer CPG. The first stimulus applied to extensor pattern formation neuron from 1 to 1.1 s (blue area) with a current of 2 nA and duration of 0.1 s is shorter than one swing–stance period. The second stimulus is applied from 2.5 to 3.5 s (orange area) with a current of 2 nA and duration of 1 s is longer than one swing–stance period. The simulation startup transients are not shown for the first 0.5 s. MN: Motoneuron; PF: Pattern formation; RG: Rhythm generation.

The result of external stimulus to the rhythm generators demonstrates that rhythmic periods can be modulated with minimal influence on the motoneuron activation amplitudes. When a positive electrical current is applied to both rhythm generator neurons (orange area in Figure 5), the period of one stride is shortened from 0.5 to 0.35 s. After the excitatory stimulus is removed, the period immediately returns to 0.5 s. When negative electrical current is applied to both rhythm generator neurons (blue area in Figure 6), the cycle period is prolonged to 0.65 s per period. The cycle period is calculated using MATLAB (MATLAB 2017b, The MathWorks, Inc., Natick, MA, USA). Each new period begins when the extensor pattern formation neuron voltage, $V_{PFext}$, crosses a threshold (i.e., $V_{PFext}=-60\;mV$ and $\frac{dV_{PFext}}{dt}>0$).

The results of the influence of external currents on the pattern formation layer is shown in Figure 6. In the first stimulus, a positive current of duration of 0.1 s (blue area in Figure 6) is applied to the extensor neuron of the pattern formation layer, which is shorter than the duration of the extensor period. This result meets the first observation from the cat fictive locomotion: the influence is limited to within the ongoing step period, and does not affect the timing of the following step. The second stimulus is a positive current applied to the extensor neuron of the pattern formation layer for a length of 1 s, which is longer than one period duration (orange area in Figure 6). When the duration of stimulus is less than one step period, the extensor period is shortened and the flexor period is prolonged after the stimulus ends. When the duration of the stimulus is longer than one step period, the rhythmic bursting of motoneurons disappears during the stimulus, but reappears on the expected next step timing without causing a phase shift. These results demonstrate that the two-layer CPG structure can reproduce the observations from fictive locomotion of decerebrate cats [13,14] and isolated mice [17].

For the intact rat hindlimb walking simulations shown in Figure 5 and Figure 6, as there is no neural connectivity between the left and right hindlimb to initiate alternating gait walking, a tonic current of 2 nA with duration of 0.5 s was injected in the extensor neuron of the left rhythm generator at the start of the simulation. The first 0.5 s, referred to as the simulation startup transient, are not shown in these figures.

### 2.3. Animal Experimental Procedure

We also performed animal experiments to collect rat motion data. The preparations are female *Rattus norvegicus* Han Wistar rats and all rats were at least 6 months old. Animal care was in accordance with German animal welfare regulations, and experimental procedures were registered with the Thuringian Committee for Animal Research (JSHK-2684-05-04/12-1). We have chosen rats as the experimental subject because their size makes them excellent for studying full body kinematics in front of our three-dimensional X-ray acquisition system. Moreover, they are easy to handle and train, and are robust against infections and heal quickly after experimental procedures.

**Figure 7:**
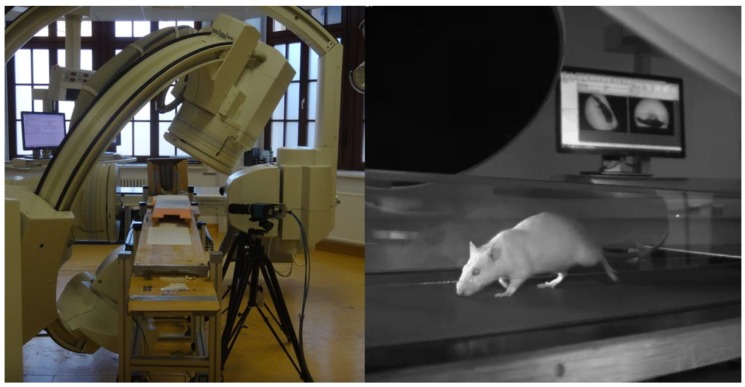
(**Left**) Workspace and Siemens Neurostar X-ray fluoroscope. (**Right**) Recording of a rat walking on the treadmill.

The motion data was collected through X-ray videography of rat walking on treadmills (Figure 7). The X-ray device (Neurostar, Siemens, Erlangen, Germany,) records at frequencies up to 2000 Hz with high resolution (1536 × 1024 dpi) high-speed cameras (SpeedCam Visario G2, Weinberger GmbH, Erlangen, Germany), enabling us to record movements precisely. Nine rats were used in total for motion capture. The treadmills (Tetra, Ilmenau, Germany) were setup at 0.5 m/s, and around 400 steps were analyzed for hard, medium, and soft belt runs. The motion data was analyzed to determine joint angle motions and to be used in SIMSCAN tools [27] as desired data to tune the simulations.

## 3. Results

### 3.1. Intact Model

To test the hypothesis that the SNS with the addition of the rhythm generator is capable of different modes of sensory control and non-resetting deletions, we ran tests to investigate if the full neuromechanical model can replicate the observed animal behaviors. To better observe the rhythm maintenance and phase shifting, the network was tuned to make the rat produce hopping motions in simulation, in which the behavior and neuron activities of the left and right hindlimbs are mirrored. To eliminate other influences, we applied each stimulus into the network with the same intensity and activation timing.

**Figure 8:**
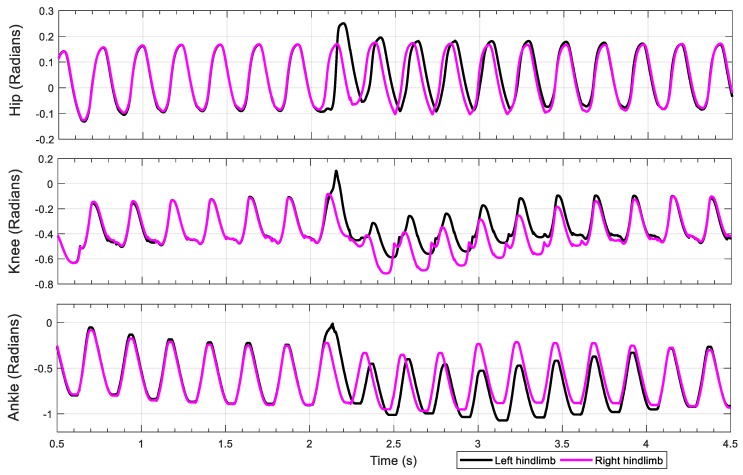
Limb joint motion during a non-resetting experiment. The stimulus is applied to the left hip pattern formation extensor neuron from 2 to 2.1 s with a current of −10 nA.

The first experiment tests for non-resetting deletions. By inhibiting the extensor neuron of the pattern formation layer (PF EXT in Figure 4C), we expected to observe non-phase-shifting on the post-inhibition rhythm. We applied −10 nA tonic stimulus to inhibit the left hip pattern formation extensor neuron from 2 to 2.1 s. Figure 8 shows the joint motions corresponding to this stimulus. The rat hindlimbs produced hopping motions until 2 s, before the inhibitory stimulus was applied. At that moment, the hip joints were still in flexion, but a short time after that the right hip joint starts extending. However, the stimulus prolonged the flexion phase of the left hindlimb, causing the left hindlimb to lag behind the master phase of the pattern generation layer. The left hip restarts extension after the stimulus ends, and generates a fast step to compensate for the delay. There is a small phase shift after the stimulus ends, but a short time later, the rhythm quickly returns to the original hopping rhythm. This brief phase shifting is caused by feedback coordinating the joint. As shown in Figure 8, the knee and the ankle joint also shift their phases accordingly to produce steady steps.

**Figure 9:**
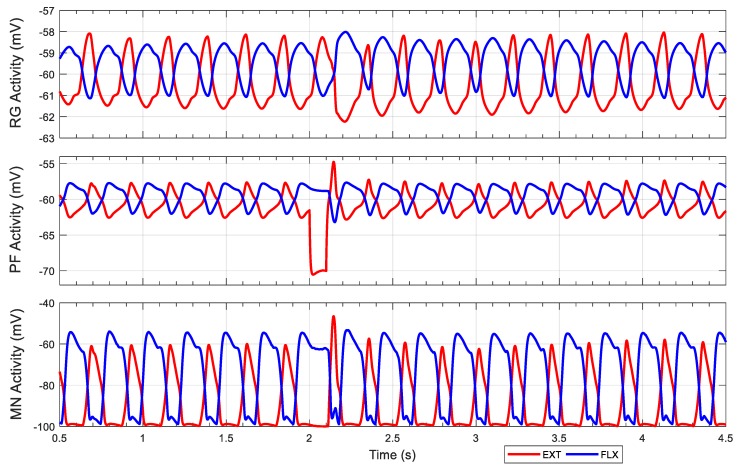
Neuron activities during non-resetting experiment. Inhibitory stimuli applied to the left hip pattern formation extensor neuron from 2 s to 2.1 s with a current of −10 nA. MN: Motoneuron; PF: Pattern formation; RG: Rhythm generation.

**Figure A1:**
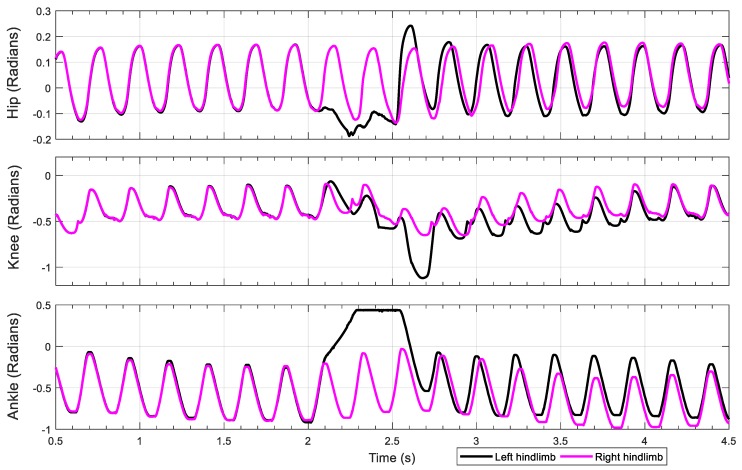
Limb joint motion during a non-resetting experiment. The stimulus is applied to the left hip pattern formation extensor neuron from 2 to 2.5 s with a current of −10 nA.

To better understand this rhythm behavior, we inspected the neuron activities of the left hindlimb. As shown in Figure 9, the extensor neuron in the pattern formation network is inhibited by the stimulus, reducing the activation time of the extensor motoneuron. The cycle timing generated by the rhythm generator is not influenced by the stimulus directly; after the stimulus ends, the extension phase is still ongoing. The pattern formation extensor neuron, driven by the rhythm generator, undergoes a swift hyperpolarization and produces an increase in neuron activation that compensates for the delay, which leads to an overshoot of extension in the hip joint as observed on Figure 8 (*t* = 2 s, top plot, in black). In addition, the rhythm generator is affected by afferent feedback for the following few cycles, which results in some phase shifting. However, the influence of the feedback is reduced by the intrinsic rhythmogenic properties of the rhythm generator, and thus the step cycle rhythm recovers. Experimental results showing longer deletions in the pattern formation layer are presented in the Appendix B (Figure A1).

**Figure 10:**
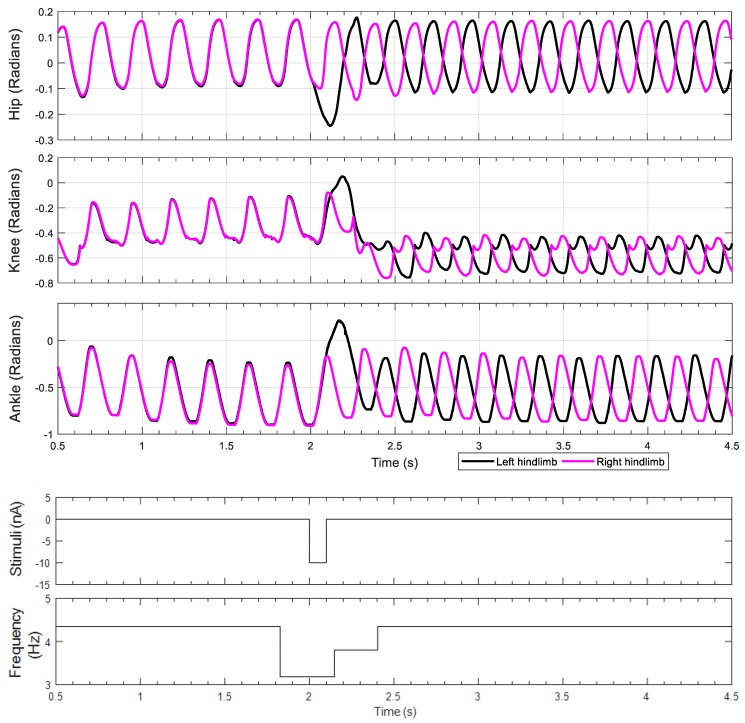
Limb joint motion for a resetting experiment. The stimulus is applied to the left hip rhythm generator extensor neuron from 2 to 2.1 s with a current of −10 nA.

Inhibiting the extensor neuron of the rhythm generator layer (RG EXT in Figure 4B) should result in permanently altering the walking rhythm. Our results confirm this; when the inhibiting stimulus is applied to the extensor neuron of the rhythm generator, the rat produces a prolonged stance phase in the left hindlimb (Figure 10, *t* = 2 s, top plot, in black), and the walking rhythm is permanently altered. This switches the gait from hopping to alternating stepping, despite there being no neural connections between the hindlimbs. Another interesting result of this change in gait is that the range of motion of the knee joint changes as well due to mechanical feedback within the system.

### 3.2. Knee–Ankle Synergy

**Figure 11:**
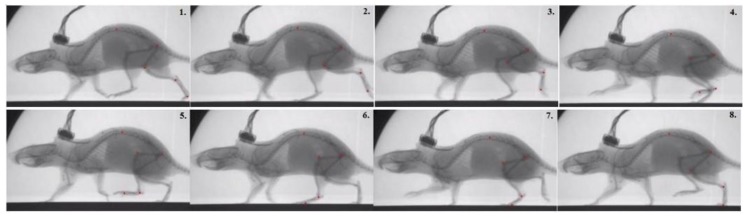
Images from X-ray video recordings of rat walking on a treadmill. Red marks are applied on the mid spine and all joints of a rat left hindlimb in order to track joint motion.

We collected kinematic and dynamic data of rats running on a treadmill. Frame by frame tracking of X-ray video recordings of these animals revealed coordination between joints. Figure 11 shows some of the frames recorded. By applying red marks on the mid spine and all joints of the rat left hindlimb, we digitally tracked the joint motion.

**Figure 12:**
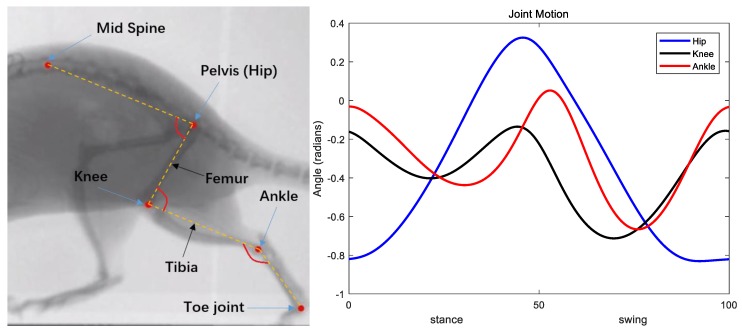
Rat left hindlimb schematic and joint motion. The ankle and the knee motion rhythms are similar, but the timing and the magnitude are different.

During normal walking, the knee and ankle joint motion profiles are similar (i.e., both biphasic) while the hip joint motion follows its own pattern. Frame 4 to frame 5 in Figure 11 show that the hip joint is still performing flexion while the ankle and the knee begin extension. Moreover, from frame 6 to frame 7, the hip extends while the ankle and the knee flex. These observations are verified by the resulting joint rotation data extracted from these frames (Figure 12). The similarity of knee and ankle motion patterns in kinematic analysis motivated us to consider the possibility of applying the same rhythm control for both the knee and ankle joints.

In our previous neuromechanical model, all three joints were independently coordinated by feedback to match the desired timing between stance and swing phase [8]. Comparing the pattern formation neuron activities of the knee and ankle joint in that model also support our hypothesis that the knee and the ankle can share the same pattern formation network to generate steady walking behavior.

**Figure 13:**
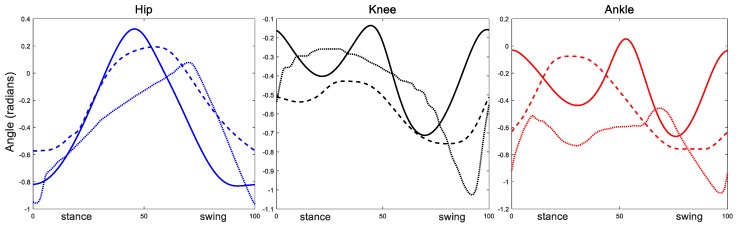
Comparison of animal joint motion profiles with simulation results. Animal data (solid lines); simulation results from this present work with the knee–ankle synergy (dashed lines); simulation results from our previous model [8] (dotted lines) with separate hip, knee and ankle pattern formation circuits.

We tuned the intact rat model to produce steady alternating stepping with an appropriate tonic noise on the left hindlimb. Simulation data collected include joint kinematics and neural activity from all neurons. Data was processed using MATLAB. The cycle period and step start time were calculated using the same method used in the deafferented model. Joint motions in each cycle were manipulated by interpolation and the mean function was used to get characteristic kinematic data. The comparison of animal joint motions with simulation characteristic kinematic data (Figure 13) shows some similarities in the hip and knee motions, but there are differences. The ankle joint shows the most difference; it is out of phase with the animal data in the stance phase. The core of the SIMSCAN tools used to tune the network is the penalty method of nonlinear optimization. The first priority is the activity of motoneurons. After that, kinematic profiles were taken into consideration with the period and height of the peaks given more weight than the mean squared error between the model and the animal joint angles. Also, the simulated muscle properties in this model need to be tuned to mimic the actual rat biomechanics. Therefore, we believe the joint motion errors are understandable. Most importantly, the simulation produced steady walking, which enabled the resetting and non-resetting experiments to be performed, which is the primary purpose of this work.

## 4. Discussion

### 4.1. Neural Control Adaptations

In this work, we present improvements to our previous neuromechanical model for rat locomotion [8] for the purpose of performing resetting and non-resetting experiments. First, we implemented a two-layer CPG structure, which provides the network with a rhythm generation layer. This addition enables more robust control of the stepping phase. This could be advantageous for stabilizing a legged robot controlled by a SNS, such as that reported by Hunt et al. [9]. The second improvement was the establishment of a simple muscle synergy, which coordinates the ankle and knee motion by using the same pattern formation layer to activate both joints. This is an important first step towards implementing a more sophisticated, leg-wide synergy-based control system, which is an increasingly accepted idea in motor control [20,22,23,24,30].

Resetting and non-resetting deletion experiments revealed that our improved neural model is capable of handling different sensory control tasks by separately adjusting the step cycle timing and motoneuron activation as known to occur in animals. This is in agreement with the results of the biomechanical cat model proposed by Markin and colleagues [20,22] and the neuronal behaviors of our simulation are similar to the mouse model by Zhong et al. [17]. However, we found that the rhythm generator did not perfectly match the non-resetting deletions as demonstrated in previous neural modeling studies [13]. For example, the joint angle performs an overshoot after the stimulus is applied. Also, during the fictive motion, the stepping phase returned immediately after the perturbation ended [13,20]. However, in our simulation, the stepping rhythm did not reset immediately, but reset slowly over time. This difference between our simulation and fictive motion shows the importance of physics-based mechanical modeling and sensory feedback in neural modeling studies. This mechanism could be responsible for gait stability—ensuring that local stability control takes preference over gait control, while gait control is maintained over time. Another possible explanation is that the slow recovery of stepping rhythm might be the mechanical entrainment from the contralateral hindlimb, which modifies the forces on the hindlimb in question, and not necessarily a product of solely the presence of sensory feedback in the model. Another example of mechanical entrainment is the joint range of motion change during gait switching. Our results show that both hindlimbs are affected by the stimulus, even though the stimulus is only applied to one hindlimb. The range of motion changed significantly when the perturbation occurred, especially for the knee joint, as it is sufficient to change the center of mass to balance the disturbance. The comparison of mechanical versus sensory feedback influences on coordination and gait is an interesting topic to investigate further.

The inclusion of the rhythm generator circuit expands our previous SNS to a two-layer CPG in which the top layer controls timing, and the second layer controls muscle force. The network can now handle different modes of sensory control to more effectively manage gait control. First, timing signals from other hindlimb can impact the rhythm generation (timing) layer, while force signals from within the hindlimb can be used to impact the pattern formation (magnitude) layer. Second, descending commands from the CNS can be used to interface with the rhythm generation layer, changing the rhythmic timing to change gaits without affecting muscle force generation. Descending commands from the vestibular system, however, may impact the pattern formation layer, to change the ground reaction force. Additional experiments are needed to explore how different types of descending commands from the brain to the CPG can make walking control more robust.

As stated in the Introduction, there are several differences between our model and Makin’s work. Markin’s computational circuits [20] are constructed from compartment models with multiconductance channels. Our SNS network is built on the dynamics of a leaky integrator [27], thus we focused on how signals propagate through the network, and how individual neurons activate, deactivate, and contribute to network behavior. However, it should be noted that there are fundamental similarities between the depolarization of a leaky integrator, and the firing frequency of a spiking neuron [31]. Another difference is in muscle modeling. While both models use Hill-type muscle models, they differ in the length tension equation and the way in which afferent feedback is calculated. Markin’s biomechanical model is based on the work by Prilutsky et al. [32], and ours simulates muscles using the model proposed by Cofer et al. [28]. Another difference is how the models interact with the environment. In Markin’s model, the ground reaction viscoelastic forces are computed as a function of velocity and displacement of the leg endpoint during stance. However, in our model, there is friction between a stance foot and the ground. Our model is capable of propelling the simulated animal forward versus walking on a belt. However, the primary difference that distinguishes the present work is that we performed different experiments with our rat model and described exclusive results.

### 4.2. Knee–Ankle Synergy

After implementing a knee–ankle synergy, our SNS successfully produced steady hopping (Figure 8) and alternating stepping without interlimb neural connections (Figure 10). Since the knee and the ankle share the same pattern formation layer, the complexity of the neural system was reduced. As shown in Figure 13, the hip and knee joint motion patterns for walking generally match the animal behavior patterns with differences in total excursion and timing. Furthermore, our new results appear improved in comparison to our previous model.

Kinematics between the simulation and those obtained by experimental data with the animal do not match well because of some limitations and approximations of the model. However, when making the simplifications necessary for neuromechanical simulations, there will always be difficulty in matching experimental data. For example, in the work by Markin et al. [20], they tuned the weight of synaptic connection to match the cat walking kinematics, but the muscle activity does not always match well. In our model, the ankle joint also does not match well with the animal data, and the synergy model shows worse matching than our previous model. However, we do not believe these results are surprising for several reasons. First, neither model was optimized to produce specific kinematics, so it is not clear if each individual model would be able to match the kinematics better. Additionally, the poor match for both models is likely the result of simplifications in the biomechanical model. That is, the reduction in degrees of freedom and number of muscles limits how the biomechanical model interacts with the ground and produces torques about the joints. This mismatch with ankle joint motion is also found in other models [20]. The advantage of these simplifications is much greater simulation speed, which enables more rapid tuning of parameters in the neural network using our MATLAB toolbox SIMSCAN [27].

It is noteworthy that the presented work is useful for understanding the underlying coordination within the neural system, and not in understanding the specific activation of muscles during walking. Given enough complexity of a model, its kinematics and muscle activations can always be tuned to produce some specific motions and values. For instance, different species of quadrupeds demonstrate different kinematics, despite anatomical similarities at the skeletal, muscular, and neural levels. Despite their different walking patterns, animals can still adapt their locomotion and gait. Such flexibility suggests that feed-forward muscle activation, descending commands, and proprioceptive feedback are all used to shape motor output, motivating the continued study of hypothetical circuits that may facilitate such control. This specific work demonstrates how the nervous system can serve multiple purposes at the same time; in our case, maintaining proper interlimb coordination while maintaining stability in individual stepping limbs.

The limitations of model simplification lead to the question: Will those flaws be solved by including more details in the biomechanical model? The rat has 38 muscles in each of its hindlimbs to control seven degrees of freedom. Even though rats have many more muscles in their legs than appear necessary for locomotion, those “redundant” muscles probably perform functions that are not yet understood. We expect that simplified biomechanical models will fail to reproduce or reduce the efficiency of some animal postures and movements. Therefore, future work will include adding more muscles including biarticular muscles [33]. Such more complex models can compare joint angle motion profiles and how the neuromechanical model reacts to perturbations as the number of hind limb muscles is increased.

Future work could also extend the network with additional structures or different connectivity in the pattern formation layer to establish time-varying synergies inside the neural network. This process could lead to insights in to how to design time-varying synergies for more complicated muscular systems, motivating the use of a more sophisticated full muscle structure in future models. In the model by Hart and Giszter [25], temporally coordinated (but not necessarily synchronous) drives are supplied to groups of muscles [23,25]. The time-varying synergies show more details of how muscles co-activate with time delays during locomotion. However, directly applying time-varying synergies will result in redundancy. As our work [33] shows, most of the muscles in any muscle group are active at the same time.

The results of the work by Markin and colleagues [20,22] has revealed that muscles which are active at the same time can share the same circuits in the pattern formation layer. Those works provide a powerful tool to help understand rat muscle synergies. In addition, adjusting the conductance of synapses between the pattern formation layer and motoneurons could allow those muscles to produce different force amplitude. It is possible to modulate the time delays between different muscle groups by setting up a dynamical interneuron or interneurons between the rhythm generator and pattern formation networks. The modularity of the SNS with two-layer CPGs makes it possible to implement both amplitude and time-varying synergies at the same time and reduce the complexity of the control problem.

Extending purely neurological models to include biomechanical components with sensory feedback enables studying additional questions regarding locomotion. For instance, one can investigate the influence of different kinds of disturbances on locomotion. In the present study, we only tested how this biomechanical model reacts to one kind of stimulus. There are many other types of stimuli that can be tested in a more complete neuromechanical model of the rat, such as stepping over obstacles, walking along an incline or decline, and stepping into a hole in the ground (postponed ground touch). There exist studies of how rats [34,35] and cats [36,37] react to these types of disturbances. In future work, we will test and expand our SNS to encompass rat-like behaviors in response to these perturbations [9].

## Appendix A

**Table A1:** Neural parameters.

Neuron	$C_{m}\;(\mathrm{nF})$	$G_{m}\;(\mu S)$	$E_{r}$ (mV)	$G_{Na}\;(\mu S)$	$A_{h}$	$S_{h}$	$E_{h}$ (mV)	$\tau_{h}$ (ms)	$A_{m}$	$S_{m}$	$E_{m}$ (mV)	$\tau_{m}$ (ms)
RG	5	1	−60	1.5	0.5	−0.6	−60	0.35	1	0.2	−40	2
PF	5	1	−60	1.5	0.5	−0.6	−60	0.35	1	0.2	−40	2
MN	5	1	−100	0	-	-	-	-	-	-	-	-
IN	5	1	-60	0	-	-	-	-	-	-	-	-

**Table A2:** Synapse parameters.

Synapse	g (μS)	$g_{ext}$ (μS)	$g_{flx}$ (μS)	$E_{s}$ (mV)	$E_{lo}$ (mV)	$E_{hi}$ (mV)
RG to IN	2.749	-	-	−40	−60	−25
IN to RG	2.749	-	-	−70	−60	−25
Between RG	0.1	-	-	−40	−65	−40
PF to IN	2.749	-	-	−40	−60	−25
IN to PF	2.749	-	-	−70	−60	−25
RG to PF	0.1	-	-	−40	−60	−40
PF to MN	-	-	-	−10	−60	−50
Hip	-	2.565	3.632	-	−60	−40
Knee	-	4.93	1.516	-	−60	−40
Ankle	-	4.054	4.522	-	−60	−40
PF to Ia	0.5	-	-	−40	−60	−55
Between Ia	0.5	-	-	−70	−60	−40
Ia to MN	2	-	-	−100	−60	−40
MN to RE	0.5	-	-	−40	−100	−10
Between R	0.5	-	-	−70	−60	−40
R to MN	0.5	-	-	−100	−60	−40
R to Ia	0.5	-	-	−70	−60	−40
